# Clinical Screening for COVID-19 in Asymptomatic Patients With Cancer

**DOI:** 10.1001/jamanetworkopen.2020.23121

**Published:** 2020-09-29

**Authors:** Manish A. Shah, Sebastian Mayer, Francie Emlen, Evan Sholle, Paul Christos, Melissa Cushing, Manuel Hidalgo

**Affiliations:** 1 Department of Medicine, Division of Hematology and Medical Oncology, Weill Cornell Medicine, New York, New York; 2 Oncology Service Line, NewYork-Presbyterian Hospital, New York; 3 Department of Information Technologies and Services, Weill Cornell Medicine, New York, New York; 4 Department of Medicine, Division of Biostatistics and Epidemiology, Weill Cornell Medicine, New York, New York; 5 Department of Anatomic and Clinical Pathology, Weill Cornell, New York, New York

## Abstract

This quality improvement study examines the efficacy of a repeated testing protocol for coronavirus disease 2019 (COVID-19) among patients with cancer.

## Introduction

As the coronavirus disease 2019 (COVID-19) pandemic continues across the United States, a critical issue for practicing oncologists is how to continue cancer care while protecting patients.^1^ To continue care for patients while also minimizing exposure to health care staff, the Weill Cornell Medicine Division of Hematology and Medical Oncology created separate units for patients with confirmed COVID-19, patients with symptomatic but unconfirmed COVID-19, and asymptomatic patients with no known high-risk COVID-19 exposures.^2^ Patients were contacted prior to their appointment and triaged based on their COVID-19 risk status. To understand the success of our clinical screening and triaging procedures, we implemented a severe acute respiratory syndrome coronavirus 2 (SARS-CoV-2) testing protocol in asymptomatic patients who required cancer care across the Division of Hematology and Medical Oncology.

## Methods

This quality improvement study, approved by Weill Cornell Medicine, follows the Standards for Quality Improvement Reporting Excellence (SQUIRE) reporting guideline. Informed consent was waived because data were deidentified, per institutional policy.

Patients were considered asymptomatic if they had no recent fever (defined as body temperature ≥100.5 °F for ≥5 days); no COVID-19 symptoms, which included cough, headache, loss of taste, and shortness of breath^3^; or high risk exposure (eg, contact with an individual with confirmed COVID-19, nursing home stay, or hospitalization) within 14 days. Diagnosis of COVID-19 was confirmed using SARS-CoV-2 nasal swab polymerase chain reaction (PCR) testing (Cobas 6800; Roche Diagnostics) and SARS-CoV-2 IgM and IgG serological tests (Pylon 3D; ET HealthCare).^4^ Patients were retested every 1 to 2 weeks. Rates of positive SARS-CoV-2 PCR and COVID-19 serological tests and 95% CIs are reported, using the 2-sided Clopper-Pearson (exact) 95% CI. Statistical analyses were conducted using Stata statistical software version 13.1 (StataCorp).

## Results

From April 30 through June 2, 2020, we performed 621 SARS-CoV-2 PCR tests on 537 asymptomatic patients (272 [50.7%] men) with hematologic or solid tumor malignant neoplasms (Table 1). Our patients were geographically representative of New York, New York, and surrounding areas, and more than 90% of patients were receiving active cytotoxic or targeted therapy. The rate of SARS-CoV-2 positivity was 0.64% (95% CI, 0.18%-1.64%). This includes 84 patients who had repeated SARS-CoV-2 tests, all of which were negative. Only 4 asymptomatic patients had test results positive for COVID-19, 2 each for hematologic and solid tumor neoplasms. We also performed serological tests from May 18 to June 2, 2020, on 238 asymptomatic patients. The rate of COVID-19 prior exposure in our asymptomatic cancer population was 4.23% (95% CI, 2.05%-7.65%) (Table 2). Notably, during this period, the SARS-CoV-2 PCR positivity rate in New York City in early May was 20%.^5^

**Table 1.** Demographic Characteristics of Asymptomatic Testing Population

Characteristic	No. (%) (N = 537)
Men	272 (50.7)
Race	
Black	75 (14.0)
Asian	47 (8.8)
White	346 (64.4)
Other^a^	24 (4.5)
American Indian	2 (0.4)
Unknown	43 (8.0)
Borough	
Bronx	31 (5.7)
Brooklyn	113 (21.0)
Manhattan	145 (27.0)
Queens	88 (16.4)
Staten Island	17 (3.2)
Tri-state	143 (26.6)
Hematologic characteristics	
No.	299
Lymphoma	57 (19)
Leukemia	71 (23.7)
BMT	72 (24.1)
Myeloma	99 (33)
Solid tumor	
No.	238
Breast	52 (21.8)
Lung	31 (13.0)
Upper GI tract or hepatobiliary	68 (28.6)
Lower GI tract	65 (27.3)
Other^b^	22 (9.2)

Abbreviations: GI, gastrointestinal; BMT, bone marrow transplant.

^a^ Other combination or not described.

^b^ Includes sarcoma, melanoma, head and neck, or neuroendocrine.

**Table 2.** SARS-CoV-2 PCR and COVID-19 Serology Testing in Clinically Screened Asymptomatic Patient Populations

Tumor type	SARS-CoV-2 PCR tests			COVID-19 serology tests			
	No.		Positive rate (95% CI), %	No.			Positive rate (95% CI), %
	Total	Positive result		Total	Positive result^a^	Indeterminate result^a^	
Solid tumor	270	2	0.74 (0.10-2.65)	131	3	8	2.29 (0.48-6.55)
Hematologic malignant neoplasm	351	2	0.57 (0.07-2.04)	105	7	3	6.67 (2.72-13.25)
Total	621	4	0.64 (0.18-1.64)	236	10	11	4.23 (2.05-7.65)

Abbreviations: COVID-19, coronavirus disease 2019; PCR, polymerase chain reaction; SARS-CoV-2, severe acute respiratory syndrome coronavirus 2.

^a^ Positive was defined as value 1 or greater, and intermediate was defined as the mean index value plus 3 × SD divided by the instrument cutoff.

## Discussion

The findings of this quality improvement study have several important implications. While it was clearly prudent to postpone elective procedures and delay care when possible, it is also clear that the risk of COVID-19 is likely to persist for some time. Cancer care will need to continue, given that further delays will lead to significantly worse outcomes for individual patients^6^ and other health care crises. We observed that the rate of past infection in our clinically screened asymptomatic cancer population was exceedingly low, at approximately 4%, and the rate of SARS-CoV-2 PCR positivity was less than 1%. This is in contrast to the COVID-19 prevalence in New York City at that time, which was close to 20%. While we do not know the reason for this low prevalence rate, it is likely that these highly motivated patients abided to social distancing recommendations, masking, and hygiene. We found that patients whose test results were negative for COVID-19 could receive chemotherapy without increasing the risk of contracting the disease, further bolstering the argument that clinicians may resume anticancer therapy in asymptomatic patients. Our quality initiative study was limited by the relatively small sample size of our patients in racial/ethnic minority groups and relatively short patient follow-up. However, we found that with proper patient education and clinical screening for symptoms or viral exposure, we maintained the outpatient clinical arena largely free of SARS-CoV-2. These data provide some reassurance to health care workers and patients that oncological treatment may safely continue.
